# Morpholine–4-nitro­phenol (1/2)

**DOI:** 10.1107/S1600536812047174

**Published:** 2012-11-24

**Authors:** Srinivasan Muralidharan, Yechuri Vidyalakshmi, Thothadri Srinivasan, Rengasamy Gopalakrishnan, Devadasan Velmurugan

**Affiliations:** a Department of Physics, Anna University, Chennai 600 025, India; b Centre of Advanced Study in Crystallography and Biophysics, University of Madras, Guindy Campus, Chennai 600 025, India

## Abstract

In the title adduct, 2C_6_H_5_NO_3_·C_4_H_9_NO, the morpholine ring adopts a chair conformation. The dihedral angle between the two nitro­phenol rings is 69.47 (9)°. The nitro groups attached to the benzene rings make dihedral angles of 3.37 (16) and 3.14 (13)° in the two mol­ecules of nitro­phenol. The crystal structure is stabilized by N—H⋯O, O—H⋯N and O—H⋯O hydrogen bonds and further consolidated by C—H⋯O inter­actions, resulting in a three-dimensional network.

## Related literature

For the biological activity and synthesis of 4-(4-nitro­phen­yl)–morpholine derivatives, see: Wang *et al.* (2010 ▶). For a related structure, see: Wang *et al.* (2012 ▶).
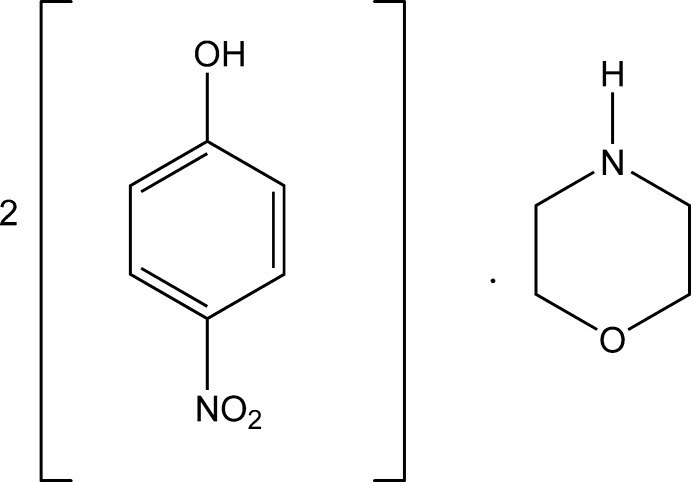


## Experimental

### 

#### Crystal data

2C_6_H_5_NO_3_·C_4_H_9_NO
*M* *_r_* = 365.34
Monoclinic, 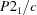

*a* = 18.0381 (7) Å
*b* = 5.5673 (2) Å
*c* = 17.4910 (7) Å
β = 91.606 (3)°
*V* = 1755.82 (12) Å^3^
*Z* = 4
Mo *K*α radiation
μ = 0.11 mm^−1^
*T* = 293 K
0.35 × 0.30 × 0.25 mm

#### Data collection

Bruker SMART APEXII area-detector diffractometer
Absorption correction: multi-scan (*SADABS*; Bruker, 2008 ▶) *T*_min_ = 0.963, *T*_max_ = 0.973
16662 measured reflections
4354 independent reflections
2987 reflections with *I* > 2σ(*I*)
*R*_int_ = 0.027

#### Refinement

*R*[*F* ^2^ > 2σ(*F* ^2^)] = 0.052
*wR*(*F* ^2^) = 0.151
*S* = 1.05
4354 reflections
239 parameters
H atoms treated by a mixture of independent and constrained refinement
Δρ_max_ = 0.45 e Å^−3^
Δρ_min_ = −0.30 e Å^−3^

### 

Data collection: *APEX2* (Bruker, 2008 ▶); cell refinement: *SAINT* (Bruker, 2008 ▶); data reduction: *SAINT*; program(s) used to solve structure: *SHELXS97* (Sheldrick, 2008 ▶); program(s) used to refine structure: *SHELXL97* (Sheldrick, 2008 ▶); molecular graphics: *ORTEP-3* (Farrugia, 2012 ▶); software used to prepare material for publication: *SHELXL97* and *PLATON* (Spek, 2009 ▶).

## Supplementary Material

Crystal structure: contains datablock(s) global, I. DOI: 10.1107/S1600536812047174/pv2605sup1.cif

Structure factors: contains datablock(s) I. DOI: 10.1107/S1600536812047174/pv2605Isup2.hkl

Additional supplementary materials:  crystallographic information; 3D view; checkCIF report

## Figures and Tables

**Table 1:** Hydrogen-bond geometry (Å, °)

*D*—H⋯*A*	*D*—H	H⋯*A*	*D*⋯*A*	*D*—H⋯*A*
N3—H3*B*⋯O7^i^	0.77 (2)	2.46 (2)	3.000 (2)	129 (2)
N3—H3*B*⋯O4^ii^	0.77 (2)	2.36 (2)	3.032 (2)	147 (2)
C14—H14*B*⋯O4^ii^	0.97	2.55	3.322 (3)	136
O3—H3*A*⋯O6	0.82	1.77	2.590 (2)	173
O6—H6*A*⋯N3	0.82	1.93	2.607 (2)	140
C6—H6⋯O2^iii^	0.93	2.53	3.424 (3)	161
C14—H14*A*⋯O5^iv^	0.97	2.49	3.403 (3)	157
C15—H15*B*⋯O1^v^	0.97	2.48	3.400 (3)	159
C16—H16*B*⋯O2^vi^	0.97	2.56	3.441 (3)	152

Symmetry codes: (i) 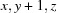; (ii) 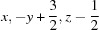; (iii) 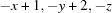; (iv) 
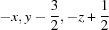
; (v) 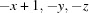; (vi) 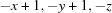.

**Fig. 1.**
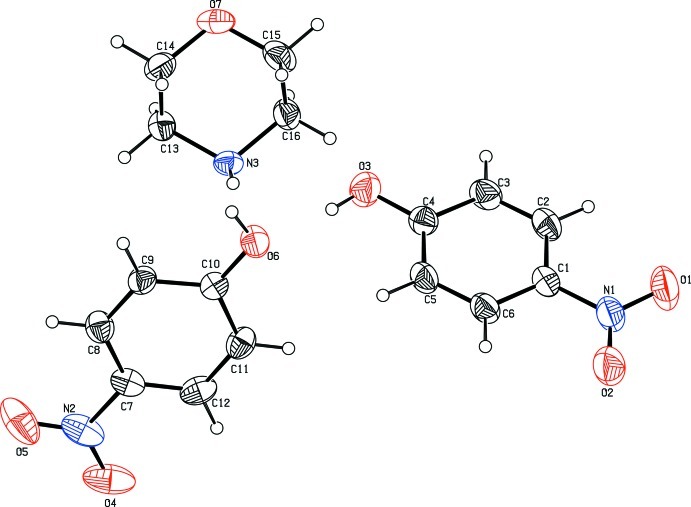
The molecular structure of the title compound, showing displacement ellipsoids drawn at the 30% probability level. H atoms are presented as small spheres of arbitrary radius.

**Fig. 2.**
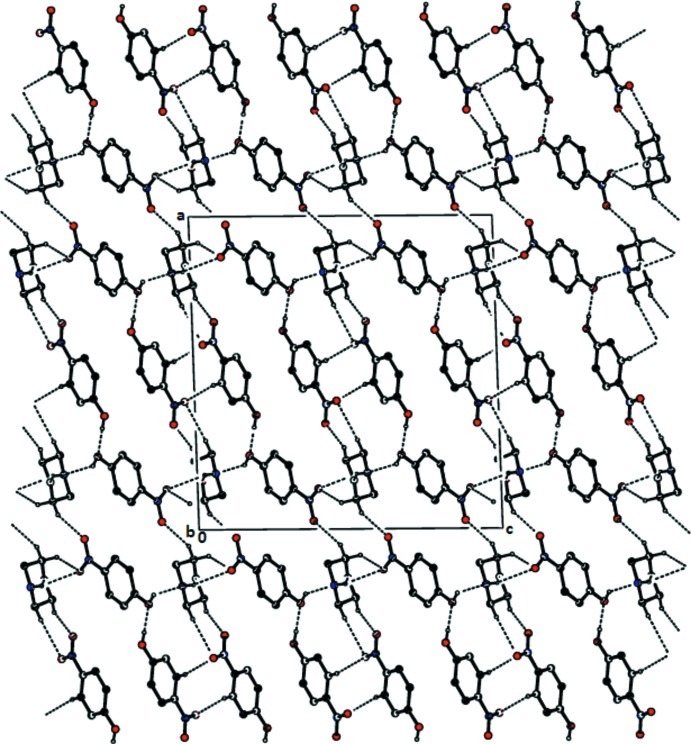
A view of the hydrogen bonds (dotted lines) in the crystal structure of the title compound. H atoms non-participating in hydrogen- bonding were omitted for clarity.

## supplementary crystallographic information

### Comment

4-(4-Nitrophenyl)morpholine derivatives are of great importance due to their anticancer activity (Wang *et al.*, 2010). The title adduct is a key intermediate in the synthetic investigations of antitumor drugs. We report the preparation and crystal structure of the title adduct in this paper.

In the title adduct (Fig. 1), the morpholine ring adopts a chair conformation. The dihedral angle between the two nitrophenol rings is 69.47 (9)°. The nitro group attached with the benzene ring (C1–C6) makes a dihedral angle of 3.37 (16)°, while the other nitro group attached with the other benzene ring (C7–C12) makes a dihedral angle of 3.14 (13)°. The crystal structure is stabilzied by intermolecular interactions of the types N—H···O and O—H···O and further consolidated by C—H···O intermolcular hydrogen bonds (Tab. 1 & Fig. 2) resulting in a 3-dimensional network.

### Experimental

Morpholine and 4-Nitrophenol were taken in equimolar (1:1) ratio using ethanol as solvent. The solution was filtered in a clean beaker and optimally closed. The solution was kept at room temperature. After two weeks, a product was obtained which was subsequently recrystallised from ethanol resulting in yellow coloured crystals suitable for X-ray diffraction.

### Refinement

All C-bound H atoms were positioned geometrically and refined using a riding model, with C—H = 0.93 and 0.97 Å, for aryl and methylene H-atoms, respectively. The hydroxyl H-atoms were included at geometrically calculated positions with O—H = 0.82 Å. The H-atom bonded to N3 was located from a difference map and allowed to refine freely. The *U*_iso_(H) were allowed at 1.5*U*_eq_(O) or 1.2*U*_eq_(C/N).

### Crystal data

**Table:**

2C_6_H_5_NO_3_·C_4_H_9_NO	*F*(000) = 768
*M**_r_* = 365.34	*D*_x_ = 1.382 Mg m^−^^3^
Monoclinic, *P*2_1_/*c*	Mo *K*α radiation, λ = 0.71073 Å
Hall symbol: -P 2ybc	Cell parameters from 4354 reflections
*a* = 18.0381 (7) Å	θ = 1.1–28.4°
*b* = 5.5673 (2) Å	µ = 0.11 mm^−^^1^
*c* = 17.4910 (7) Å	*T* = 293 K
β = 91.606 (3)°	Block, colourless
*V* = 1755.82 (12) Å^3^	0.35 × 0.30 × 0.25 mm
*Z* = 4	

### Data collection

**Table:**

Bruker SMART APEXII area-detector diffractometer	4354 independent reflections
Radiation source: fine-focus sealed tube	2987 reflections with *I* > 2σ(*I*)
Graphite monochromator	*R*_int_ = 0.027
ω and φ scans	θ_max_ = 28.4°, θ_min_ = 1.1°
Absorption correction: multi-scan (*SADABS*; Bruker, 2008)	*h* = −24→24
*T*_min_ = 0.963, *T*_max_ = 0.973	*k* = −7→7
16662 measured reflections	*l* = −23→22

### Refinement

**Table:**

Refinement on *F*^2^	Primary atom site location: structure-invariant direct methods
Least-squares matrix: full	Secondary atom site location: difference Fourier map
*R*[*F*^2^ > 2σ(*F*^2^)] = 0.052	Hydrogen site location: inferred from neighbouring sites
*wR*(*F*^2^) = 0.151	H atoms treated by a mixture of independent and constrained refinement
*S* = 1.05	*w* = 1/[σ^2^(*F*_o_^2^) + (0.0638*P*)^2^ + 0.4643*P*] where *P* = (*F*_o_^2^ + 2*F*_c_^2^)/3
4354 reflections	(Δ/σ)_max_ < 0.001
239 parameters	Δρ_max_ = 0.45 e Å^−^^3^
0 restraints	Δρ_min_ = −0.30 e Å^−^^3^

### Special details

**Table:**

Geometry. All esds (except the esd in the dihedral angle between two l.s. planes) are estimated using the full covariance matrix. The cell esds are taken into account individually in the estimation of esds in distances, angles and torsion angles; correlations between esds in cell parameters are only used when they are defined by crystal symmetry. An approximate (isotropic) treatment of cell esds is used for estimating esds involving l.s. planes.
Refinement. Refinement of F^2^ against ALL reflections. The weighted R-factor wR and goodness of fit S are based on F^2^, conventional R-factors R are based on F, with F set to zero for negative F^2^. The threshold expression of F^2^ > 2sigma(F^2^) is used only for calculating R-factors(gt) etc. and is not relevant to the choice of reflections for refinement. R-factors based on F^2^ are statistically about twice as large as those based on F, and R- factors based on ALL data will be even larger.

### Fractional atomic coordinates and isotropic or equivalent isotropic displacement parameters (Å^2^)

**Table:**

	*x*	*y*	*z*	*U*_iso_*/*U*_eq_	
C1	0.53902 (9)	0.5777 (3)	0.10103 (10)	0.0565 (4)	
C2	0.55081 (10)	0.3693 (4)	0.14223 (11)	0.0648 (5)	
H2	0.5982	0.3048	0.1474	0.078*	
C3	0.49232 (10)	0.2585 (4)	0.17542 (12)	0.0659 (5)	
H3	0.5001	0.1191	0.2038	0.079*	
C4	0.42092 (10)	0.3533 (3)	0.16694 (10)	0.0560 (4)	
C5	0.41037 (10)	0.5620 (3)	0.12451 (11)	0.0616 (5)	
H5	0.3630	0.6260	0.1183	0.074*	
C6	0.46899 (10)	0.6747 (4)	0.09170 (11)	0.0628 (5)	
H6	0.4617	0.8148	0.0635	0.075*	
C7	0.13017 (10)	0.8918 (3)	0.31527 (9)	0.0553 (4)	
C8	0.09076 (10)	0.7063 (4)	0.28005 (10)	0.0588 (4)	
H8	0.0404	0.6875	0.2884	0.071*	
C9	0.12639 (9)	0.5524 (3)	0.23317 (10)	0.0539 (4)	
H9	0.0996	0.4294	0.2093	0.065*	
C10	0.20240 (9)	0.5739 (3)	0.21978 (9)	0.0469 (4)	
C11	0.24071 (10)	0.7647 (4)	0.25620 (10)	0.0585 (4)	
H11	0.2911	0.7845	0.2483	0.070*	
C12	0.20488 (11)	0.9213 (3)	0.30297 (10)	0.0624 (5)	
H12	0.2308	1.0470	0.3264	0.075*	
C13	0.11073 (9)	0.0465 (3)	0.07932 (11)	0.0572 (4)	
H13A	0.0692	0.1572	0.0801	0.069*	
H13B	0.1164	−0.0247	0.1298	0.069*	
C14	0.09471 (11)	−0.1474 (3)	0.02155 (12)	0.0629 (5)	
H14A	0.0513	−0.2372	0.0364	0.076*	
H14B	0.0839	−0.0750	−0.0280	0.076*	
C15	0.21931 (12)	−0.1788 (4)	−0.00757 (13)	0.0739 (6)	
H15A	0.2090	−0.1040	−0.0568	0.089*	
H15B	0.2600	−0.2907	−0.0134	0.089*	
C16	0.24145 (10)	0.0101 (3)	0.04962 (11)	0.0594 (5)	
H16A	0.2553	−0.0654	0.0979	0.071*	
H16B	0.2841	0.0973	0.0317	0.071*	
N1	0.60131 (9)	0.6983 (4)	0.06695 (11)	0.0732 (5)	
N2	0.09282 (13)	1.0491 (3)	0.36639 (9)	0.0775 (5)	
O7	0.15556 (8)	−0.3055 (2)	0.01578 (9)	0.0733 (4)	
O1	0.66276 (8)	0.6053 (4)	0.07188 (11)	0.1022 (6)	
O2	0.59056 (10)	0.8873 (4)	0.03293 (13)	0.1106 (7)	
O3	0.36561 (7)	0.2375 (3)	0.20120 (9)	0.0792 (4)	
H3A	0.3265	0.3093	0.1929	0.119*	
O4	0.12927 (12)	1.2119 (3)	0.39774 (9)	0.1007 (6)	
O5	0.02664 (12)	1.0156 (4)	0.37853 (11)	0.1164 (7)	
O6	0.23640 (6)	0.4276 (2)	0.17466 (7)	0.0620 (3)	
H6A	0.2068	0.3287	0.1572	0.093*	
N3	0.17917 (8)	0.1798 (2)	0.06111 (9)	0.0483 (3)	
H3B	0.1748 (10)	0.258 (3)	0.0250 (11)	0.055 (6)*	

### Atomic displacement parameters (Å^2^)

**Table:**

	*U*^11^	*U*^22^	*U*^33^	*U*^12^	*U*^13^	*U*^23^
C1	0.0503 (9)	0.0618 (10)	0.0574 (10)	0.0096 (8)	0.0024 (7)	−0.0051 (9)
C2	0.0521 (9)	0.0705 (12)	0.0714 (12)	0.0183 (9)	−0.0053 (8)	−0.0017 (10)
C3	0.0634 (11)	0.0604 (11)	0.0731 (13)	0.0134 (9)	−0.0087 (9)	0.0083 (10)
C4	0.0527 (9)	0.0558 (10)	0.0592 (10)	0.0030 (8)	−0.0059 (8)	−0.0037 (8)
C5	0.0475 (9)	0.0645 (11)	0.0726 (12)	0.0143 (8)	−0.0027 (8)	0.0050 (10)
C6	0.0583 (10)	0.0596 (11)	0.0708 (12)	0.0160 (8)	0.0045 (9)	0.0081 (9)
C7	0.0746 (11)	0.0519 (9)	0.0389 (8)	0.0103 (8)	−0.0055 (7)	−0.0029 (7)
C8	0.0534 (9)	0.0688 (11)	0.0541 (10)	0.0030 (8)	0.0013 (7)	−0.0060 (9)
C9	0.0514 (9)	0.0542 (9)	0.0560 (10)	−0.0084 (7)	−0.0001 (7)	−0.0106 (8)
C10	0.0504 (8)	0.0497 (8)	0.0404 (8)	−0.0022 (7)	−0.0026 (6)	0.0002 (7)
C11	0.0559 (9)	0.0685 (11)	0.0510 (10)	−0.0146 (8)	−0.0030 (7)	−0.0055 (9)
C12	0.0846 (13)	0.0552 (10)	0.0467 (9)	−0.0163 (9)	−0.0113 (9)	−0.0067 (8)
C13	0.0520 (9)	0.0584 (10)	0.0615 (10)	0.0025 (8)	0.0093 (8)	0.0002 (8)
C14	0.0641 (11)	0.0514 (10)	0.0733 (12)	−0.0119 (8)	0.0015 (9)	0.0043 (9)
C15	0.0754 (13)	0.0680 (12)	0.0786 (14)	0.0166 (10)	0.0104 (10)	−0.0227 (11)
C16	0.0511 (9)	0.0648 (11)	0.0628 (11)	0.0035 (8)	0.0099 (8)	−0.0092 (9)
N1	0.0579 (9)	0.0808 (12)	0.0816 (12)	0.0131 (8)	0.0145 (8)	0.0011 (10)
N2	0.1161 (16)	0.0702 (11)	0.0454 (9)	0.0301 (11)	−0.0100 (9)	−0.0105 (8)
O7	0.0931 (10)	0.0374 (6)	0.0891 (10)	−0.0001 (6)	−0.0031 (8)	−0.0037 (6)
O1	0.0543 (8)	0.1261 (15)	0.1269 (14)	0.0210 (9)	0.0169 (8)	0.0207 (12)
O2	0.0830 (11)	0.0936 (12)	0.1573 (18)	0.0169 (10)	0.0437 (11)	0.0417 (13)
O3	0.0595 (8)	0.0772 (9)	0.1010 (11)	−0.0002 (7)	0.0000 (7)	0.0206 (9)
O4	0.1836 (19)	0.0604 (9)	0.0576 (9)	0.0131 (11)	−0.0061 (10)	−0.0163 (7)
O5	0.1010 (14)	0.1490 (18)	0.0994 (13)	0.0500 (13)	0.0057 (11)	−0.0458 (13)
O6	0.0515 (6)	0.0723 (8)	0.0619 (7)	0.0003 (6)	−0.0010 (5)	−0.0193 (7)
N3	0.0595 (8)	0.0377 (7)	0.0476 (8)	0.0013 (6)	0.0029 (6)	0.0053 (6)

### Geometric parameters (Å, º)

**Table:**

C1—C2	1.379 (3)	C12—H12	0.9300
C1—C6	1.379 (2)	C13—N3	1.483 (2)
C1—N1	1.451 (2)	C13—C14	1.501 (3)
C2—C3	1.366 (3)	C13—H13A	0.9700
C2—H2	0.9300	C13—H13B	0.9700
C3—C4	1.396 (2)	C14—O7	1.413 (2)
C3—H3	0.9300	C14—H14A	0.9700
C4—O3	1.343 (2)	C14—H14B	0.9700
C4—C5	1.389 (3)	C15—O7	1.419 (3)
C5—C6	1.369 (3)	C15—C16	1.498 (3)
C5—H5	0.9300	C15—H15A	0.9700
C6—H6	0.9300	C15—H15B	0.9700
C7—C12	1.380 (3)	C16—N3	1.486 (2)
C7—C8	1.388 (3)	C16—H16A	0.9700
C7—N2	1.433 (2)	C16—H16B	0.9700
C8—C9	1.360 (2)	N1—O2	1.222 (2)
C8—H8	0.9300	N1—O1	1.224 (2)
C9—C10	1.403 (2)	N2—O5	1.233 (3)
C9—H9	0.9300	N2—O4	1.238 (2)
C10—O6	1.2998 (19)	O3—H3A	0.8200
C10—C11	1.409 (2)	O6—H6A	0.8200
C11—C12	1.370 (3)	N3—H3B	0.77 (2)
C11—H11	0.9300		
			
C2—C1—C6	121.24 (17)	N3—C13—C14	111.17 (14)
C2—C1—N1	119.60 (16)	N3—C13—H13A	109.4
C6—C1—N1	119.16 (17)	C14—C13—H13A	109.4
C3—C2—C1	119.50 (16)	N3—C13—H13B	109.4
C3—C2—H2	120.2	C14—C13—H13B	109.4
C1—C2—H2	120.2	H13A—C13—H13B	108.0
C2—C3—C4	120.37 (18)	O7—C14—C13	111.16 (15)
C2—C3—H3	119.8	O7—C14—H14A	109.4
C4—C3—H3	119.8	C13—C14—H14A	109.4
O3—C4—C5	123.20 (16)	O7—C14—H14B	109.4
O3—C4—C3	117.78 (17)	C13—C14—H14B	109.4
C5—C4—C3	119.02 (17)	H14A—C14—H14B	108.0
C6—C5—C4	120.76 (16)	O7—C15—C16	111.09 (16)
C6—C5—H5	119.6	O7—C15—H15A	109.4
C4—C5—H5	119.6	C16—C15—H15A	109.4
C5—C6—C1	119.10 (18)	O7—C15—H15B	109.4
C5—C6—H6	120.4	C16—C15—H15B	109.4
C1—C6—H6	120.4	H15A—C15—H15B	108.0
C12—C7—C8	120.62 (16)	N3—C16—C15	110.39 (15)
C12—C7—N2	120.17 (18)	N3—C16—H16A	109.6
C8—C7—N2	119.19 (18)	C15—C16—H16A	109.6
C9—C8—C7	119.44 (17)	N3—C16—H16B	109.6
C9—C8—H8	120.3	C15—C16—H16B	109.6
C7—C8—H8	120.3	H16A—C16—H16B	108.1
C8—C9—C10	121.85 (16)	O2—N1—O1	121.97 (19)
C8—C9—H9	119.1	O2—N1—C1	118.97 (16)
C10—C9—H9	119.1	O1—N1—C1	119.05 (19)
O6—C10—C9	121.87 (15)	O5—N2—O4	122.6 (2)
O6—C10—C11	120.85 (15)	O5—N2—C7	119.3 (2)
C9—C10—C11	117.26 (15)	O4—N2—C7	118.1 (2)
C12—C11—C10	121.06 (17)	C14—O7—C15	110.39 (14)
C12—C11—H11	119.5	C4—O3—H3A	109.5
C10—C11—H11	119.5	C10—O6—H6A	109.5
C11—C12—C7	119.76 (16)	C13—N3—C16	110.35 (13)
C11—C12—H12	120.1	C13—N3—H3B	113.2 (14)
C7—C12—H12	120.1	C16—N3—H3B	108.0 (14)
			
C6—C1—C2—C3	−1.0 (3)	C10—C11—C12—C7	0.4 (3)
N1—C1—C2—C3	178.82 (18)	C8—C7—C12—C11	−0.7 (3)
C1—C2—C3—C4	0.8 (3)	N2—C7—C12—C11	177.62 (17)
C2—C3—C4—O3	−179.62 (18)	N3—C13—C14—O7	56.0 (2)
C2—C3—C4—C5	−0.2 (3)	O7—C15—C16—N3	−57.5 (2)
O3—C4—C5—C6	179.03 (19)	C2—C1—N1—O2	−177.4 (2)
C3—C4—C5—C6	−0.4 (3)	C6—C1—N1—O2	2.4 (3)
C4—C5—C6—C1	0.3 (3)	C2—C1—N1—O1	4.0 (3)
C2—C1—C6—C5	0.4 (3)	C6—C1—N1—O1	−176.2 (2)
N1—C1—C6—C5	−179.37 (18)	C12—C7—N2—O5	−177.76 (19)
C12—C7—C8—C9	0.1 (3)	C8—C7—N2—O5	0.6 (3)
N2—C7—C8—C9	−178.17 (17)	C12—C7—N2—O4	1.0 (3)
C7—C8—C9—C10	0.6 (3)	C8—C7—N2—O4	179.30 (17)
C8—C9—C10—O6	−179.63 (17)	C13—C14—O7—C15	−60.4 (2)
C8—C9—C10—C11	−0.8 (3)	C16—C15—O7—C14	61.5 (2)
O6—C10—C11—C12	179.10 (16)	C14—C13—N3—C16	−51.7 (2)
C9—C10—C11—C12	0.3 (3)	C15—C16—N3—C13	52.3 (2)

### Hydrogen-bond geometry (Å, º)

**Table:**

*D*—H···*A*	*D*—H	H···*A*	*D*···*A*	*D*—H···*A*
N3—H3*B*···O7^i^	0.77 (2)	2.46 (2)	3.000 (2)	129 (2)
N3—H3*B*···O4^ii^	0.77 (2)	2.36 (2)	3.032 (2)	147 (2)
C14—H14*B*···O4^ii^	0.97	2.55	3.322 (3)	136
O3—H3*A*···O6	0.82	1.77	2.590 (2)	173
O6—H6*A*···N3	0.82	1.93	2.607 (2)	140
C6—H6···O2^iii^	0.93	2.53	3.424 (3)	161
C14—H14*A*···O5^iv^	0.97	2.49	3.403 (3)	157
C15—H15*B*···O1^v^	0.97	2.48	3.400 (3)	159
C16—H16*B*···O2^vi^	0.97	2.56	3.441 (3)	152

Symmetry codes: (i) *x*, *y*+1, *z*; (ii) *x*, −*y*+3/2, *z*−1/2; (iii) −*x*+1, −*y*+2, −*z*; (iv) −*x*, *y*−3/2, −*z*+1/2; (v) −*x*+1, −*y*, −*z*; (vi) −*x*+1, −*y*+1, −*z*.
